# Real-World Clinical Practice Evaluation of Tralokinumab in Atopic Dermatitis: A 52-Week Multi-Center Retrospective Study in the Basque Country

**DOI:** 10.3390/jcm14165727

**Published:** 2025-08-13

**Authors:** Rosa María Izu Belloso, Marc Juliá Manresa, Nekane Martínez Peña, Maider Pretel Irazabal, Vanesa Fatsini Blanch, Nerea Ormaechea Pérez, Manuel Pascual Ares, Juan Antonio Ratón Nieto

**Affiliations:** 1 Dermatology Department, Hospital Universitario Basurto, 48013 Bilbao, Spain; marc.juliamanresa@osakidetza.eus (M.J.M.); 96nekane.martinez@gmail.com (N.M.P.); 2 Dermatology Department, Hospital Universitario Galdakao, 48960 Galdakao, Spain; maider.pretelirazabal@osakidetza.eus; 3 Dermatology Department, Hospital Universitario Araba, 01009 Vitoria-Gasteiz, Spain; vanesa.fatsiniblanch@osakidetza.eus; 4 Dermatology Department, Hospital Universitario Donostia, 20014 Donostia, Spain; nerea.ormaecheaperez@osakidetza.eus; 5 Dermatology Department, Hospital Universitario Cruces, 48903 Barakaldo, Spain; manuel.pascualares@osakidetza.eus (M.P.A.); juanantonio.ratonnieto@osakidetza.eus (J.A.R.N.)

**Keywords:** atopic dermatitis, tralokinumab, real-world evidence, EASI-75, biologics, 52-week follow-up

## Abstract

**Background**: Tralokinumab is an anti-IL-13 monoclonal antibody approved for moderate-to-severe atopic dermatitis (AD). While pivotal trials have demonstrated its efficacy, real-world data remain limited. **Methods**: We conducted a retrospective, multi-center study in the Basque Country including 109 adults with moderate-to-severe AD treated with tralokinumab. Clinical outcomes (EASI, IGA, BSA, DLQI, and NRS-pruritus/sleep) were assessed at baseline, weeks 16, 24, and 52. **Results**: EASI-75/90/100 responses were 66%/44%/16% at week 16 and increased to 83%/70%/34% at week 52. Pruritus NRS decreased from 7.1 to 3.1 and DLQI from 17.8 to 9.0. Adverse events were uncommon, with only three cases of conjunctivitis (two discontinued). **Conclusions**: Our findings support tralokinumab as a safe and effective long-term therapy for AD in routine practice. Results were consistent with, or superior to, pivotal and other RWE studies.

## 1. Introduction

Atopic dermatitis (AD) is a chronic, relapsing inflammatory skin disease characterized by intense pruritus, eczematous lesions, and a significant impact on quality of life. Its prevalence is estimated to be around 10–20% in children and up to 3–10% in adults worldwide [1,2]. The pathophysiology of AD involves a complex interplay of genetic, immunological, and environmental factors, with type 2 inflammation playing a central role. Among key cytokines, interleukin-13 (IL-13) has been recognized as a pivotal mediator driving skin barrier dysfunction and chronic inflammation [3]. In addition to IL-13, other type 2 cytokines such as interleukin-4 (IL-4), interleukin-5 (IL-5), IL-22, and IL-31 also play important roles in the pathogenesis of AD [1]. Moreover, recent studies have emphasized the contribution of microbial dysbiosis, both at the skin and systemic level, as well as barrier defects and alarmin activation (e.g., TSLP, IL-33, IL-25), highlighting the complex immune and environmental interplay underlying the disease [4].

Tralokinumab is a fully human monoclonal antibody that specifically binds to IL-13, inhibiting its interaction with the IL-13 receptor and downstream signaling. Its efficacy and safety for moderate-to-severe AD have been demonstrated in phase III trials (ECZTRA 1, 2, and 3), showing significant improvements in Investigator’s Global Assessment (IGA), Eczema Area and Severity Index (EASI), and patient-reported outcomes, with a favorable safety profile [5,6,7].

Despite robust data from randomized controlled trials (RCTs), real-world evidence (RWE) remains crucial to validate these findings in broader, more heterogeneous patient populations and to assess long-term outcomes beyond controlled settings. Recent observational studies have begun to explore the effectiveness and safety of tralokinumab in clinical practice, reporting promising short- and medium-term results [8,9].

However, data on long-term RWE beyond 24 weeks are still limited, particularly in southern European populations. Thus, the objective of our study was to evaluate the effectiveness and safety of tralokinumab in a multi-center, real-world cohort of adult patients with moderate-to-severe AD in the Basque Country, with a follow-up of up to 52 weeks.

## 2. Materials and Methods

This is a retrospective, multi-center, real-world study conducted across five hospitals in the Basque Country (Hospital Universitario Basurto, Hospital Universitario Galdakao, Hospital Universitario Araba, Hospital Universitario Donostia, and Hospital Universitario de Cruces). The included study population are adult patients (≥18 years) with a clinical diagnosis of moderate-to-severe atopic dermatitis (AD) who initiated treatment with tralokinumab and had at least one follow-up visit after treatment initiation. Patients with incomplete clinical data or insufficient follow-up were excluded.

Objective: To evaluate the effectiveness and safety of tralokinumab treatment in patients with moderate-to-severe atopic dermatitis in a retrospective, multi-center observational study with a 52-week follow-up.

The following variables were collected: demographic characteristics (age and sex), relevant comorbidities (asthma, rhinitis, conjunctivitis, obesity, dyslipidemia, diabetes, and others), and prior treatments for AD (cyclosporine, methotrexate, azathioprine, phototherapy, dupilumab, or others). Disease severity and impact were assessed using Eczema Area and Severity Index (EASI), Investigator’s Global Assessment (IGA), Body Surface Area (BSA) involvement, Numeric Rating Scale (NRS) for pruritus and sleep disturbance, and Dermatology Life Quality Index (DLQI). Clinicians routinely record objective disease severity measures such as EASI, IGA, BSA, and patient-reported NRS for pruritus and sleep at every visit. DLQI is also part of our standard assessment. Diagnosis of moderate-to-severe AD was based on Hanifin and Rajka criteria, with severity defined as EASI ≥ 16, BSA ≥ 10%, and/or IGA ≥ 3.

These parameters were recorded at baseline and at weeks 16, 24, and 52 of follow-up. All patients received tralokinumab with an initial loading dose of 600 mg subcutaneously, followed by 300 mg every two weeks. Adjustments were allowed according to clinical judgment, but during the study period our regional protocol mandated the continuation of the approved dosing regimen (300 mg every 2 weeks) throughout the first year, without extension to every 4 weeks, regardless of early clinical response.

Concomitant use of topical corticosteroids and other topical treatments was permitted as needed. Descriptive statistics were calculated for all demographic and clinical variables, using means ± standard deviation (SD) for continuous variables and percentages for categorical variables. The Shapiro–Wilk test was used to assess the normality of quantitative variables. When normality was not met, differences in clinical scales across visits were compared using the Wilcoxon signed-rank test. For comparisons between multiple weeks, Tukey’s test was employed. A *p*-value < 0.05 was considered statistically significant. Statistical analyses and graphical representations were performed using GraphPad Prism version 9.2 (GraphPad Software, San Diego, CA, USA) and IBM SPSS Statistics version 26.0 (IBM Corp., Armonk, NY, USA).

The study was conducted in accordance with the principles of the Declaration of Helsinki and approved by the local ethics committee (Code CEIm EOM2023038, date of approval 1 March 2024).

## 3. Results

### 3.1. Baseline Demographic and Clinical Characteristics

A total of 109 patients were included in this study. The mean age was 39.4 years (SD: 16.6) and 63.3% were male and 36.7% female. Most patients had disease onset during childhood (68.8%), with the remaining 31.2% presenting in adulthood.

The most prevalent atopic comorbidities in our cohort were rhinitis (63.3%), asthma (53.2%), and conjunctivitis (22%). Other less common conditions included food allergy (18.3%), alopecia areata (2.75%), and nasal polyposis (1.8%). No cases of eosinophilic esophagitis were reported. These findings highlight the frequent association of atopic dermatitis with respiratory and allergic comorbidities, reinforcing the systemic nature of the disease.

Regarding non-atopic comorbidities, dyslipidemia was the most frequent condition, affecting 21.1% of patients. Other reported comorbidities included obesity (16.5%), diabetes (5.5%), hepatitis (2.75%), cardiovascular disease (2.75%), history of malignancy (3.7%), and HIV infection (0.9%). These findings reflect the relevance of screening for systemic comorbidities in patients with moderate-to-severe atopic dermatitis undergoing biological treatment.

In terms of disease phenotype, 74.3% of patients had a classic pattern, while 25.7% had generalized AD. The most frequently affected areas were the head and neck (67.9%), followed by the hands (31.2%) and prurigo lesions (12.8%). Regarding prior treatments, 94.5% had received cyclosporine, 34% methotrexate, 11.9% azathioprine, and 46.8% phototherapy. Notably, 78% were naïve to advanced systemic therapies, with 12.8% previously treated with dupilumab and the rest with JAK inhibitors (Upadacitinib 3.67%, Baricitinib 4.6%, and Abrocitinib 0.9%).

Of the 109 patients included, 109 attended at least one follow-up visit, 92 had two visits, and 78 completed all three follow-up visits at weeks 16, 24, and 52. The mean duration of disease symptoms since diagnosis was 21.4 ± 11.7 years. Topical corticosteroids were used by 87% of patients during the first 16 weeks, tapering to 52% by week 52, while calcineurin inhibitors and emollients were used by 32% continuously.

At baseline the mean EASI score was 24.5 (SD: 11.4), IGA 3.2 (SD: 0.7), BSA 33.9% (SD:19.7), pruritus NRS 7.1 (SD: 2.2), sleep NRS 5.2 (SD: 2.2), and DLQI 17.8 (SD: 5.7), reflecting substantial disease burden and impact on quality of life.

All results are resumed in Table 1.

### 3.2. Clinical Outcomes

At baseline patients exhibited significant disease severity and impaired quality of life, with a mean EASI score of 24.5 (SD: 11.4), BSA of 33.9% (SD: 19.7), NRS pruritus of 7.1 (SD: 2.2), NRS sleep of 5.2 (SD: 2.2), and DLQI of 17.8 (SD: 5.7). These baseline figures reflect the burden of moderate-to-severe atopic dermatitis in a real-world population prior to initiating treatment with tralokinumab.

By week 16, a rapid and clinically meaningful improvement was observed across all outcome measures. The mean EASI score dropped to 9.1 (SD: 9.3), representing a substantial reduction in objective signs of inflammation. Similarly, BSA involvement decreased to 14.0% (SD: 16.4) and patients reported relief from subjective symptoms, with NRS pruritus improving to 4.7 (SD: 2.7) and NRS sleep disturbance to 3.6 (SD: 3.1). Quality of life also improved considerably, with the mean DLQI decreasing to 11.1 (SD: 6.3), highlighting the early benefit of treatment.

At week 24, this positive trajectory was maintained. EASI remained low at 8.6 (SD: 10.2), and further reductions were seen in BSA (12.5%, SD: 19.4). Pruritus and sleep disturbances continued to improve, with NRS pruritus at 4.2 (SD: 2.9) and NRS sleep at 2.3 (SD: 2.7). DLQI further declined to 10.5 (SD: 6.1), reflecting continued gains in patient-reported quality of life.

By week 52, the therapeutic response was consolidated. The mean EASI score fell to 3.6 (SD: 3.6), approaching near-clearance in many patients. BSA was reduced to 7.0% (SD: 11.1), and both pruritus and sleep scores reached their lowest levels, at 3.3 (SD: 2.4) and 1.8 (SD: 2.5), respectively. Importantly, DLQI continued to decline, reaching 9.0 (SD: 6.0), which signified a sustained and meaningful improvement in patients’ daily functioning and overall well-being after one year of therapy.

Notably, 78% of patients were naive to advanced therapies. Among the 22% previously exposed to biologics or JAK inhibitors, response rates were similar.

Although we did not assess site-specific scores longitudinally, the high baseline prevalence of head and neck (67.9%) and hand (31.2%) involvement suggests that the observed overall clinical improvement likely extended to these challenging localizations.

**Figure 1:**
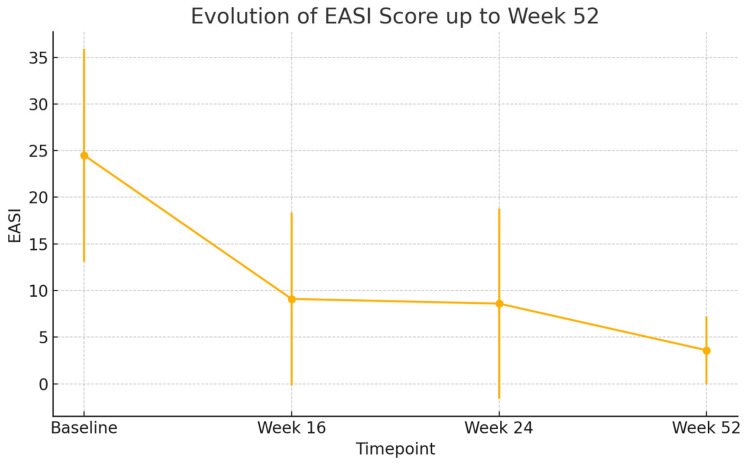
Mean EASI scores evolution. Error bars represent standard deviation (*p* < 0.001).

**Figure 2:**
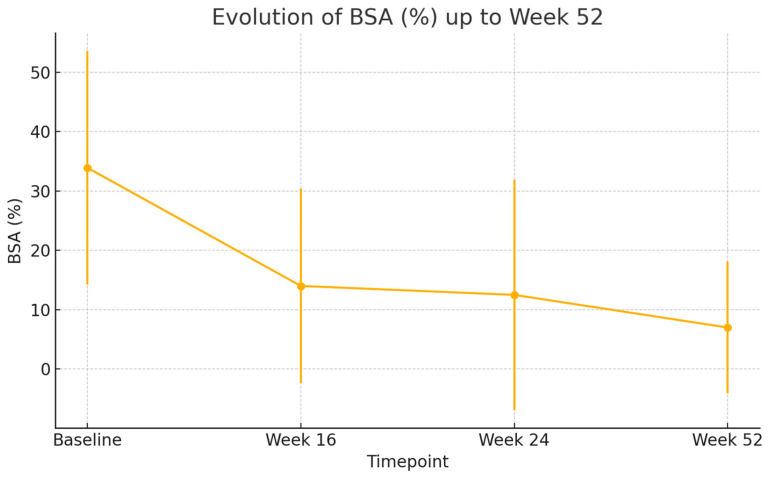
Body surface area affected by atopic dermatitis evolution. Error bars indicate standard deviation (*p* < 0.001).

**Figure 3:**
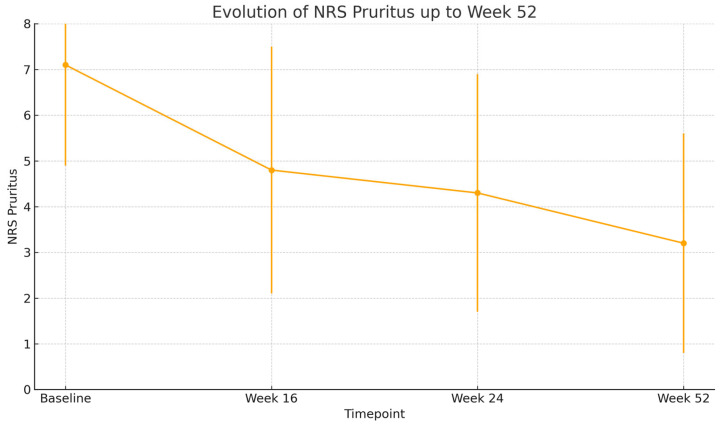
Pruritus severity, as measured by the Numerical Rating Scale (0–10) evolution. Error bars represent standard deviation (*p* < 0.001).

**Figure 4:**
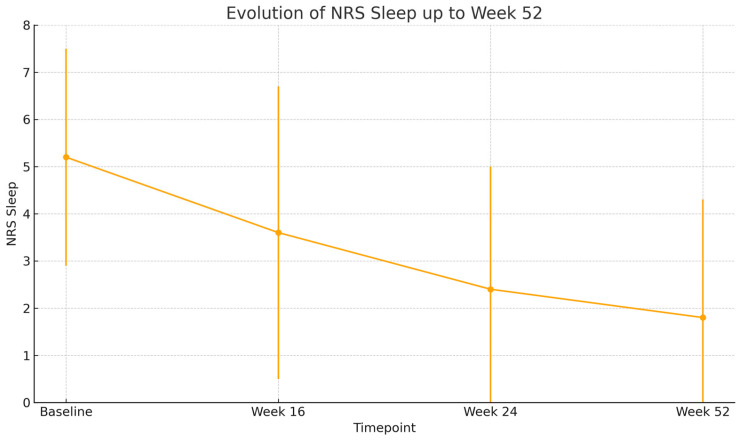
Mean NRS sleep scores evolution. Error bars show standard deviation (*p* < 0.001).

**Figure 5:**
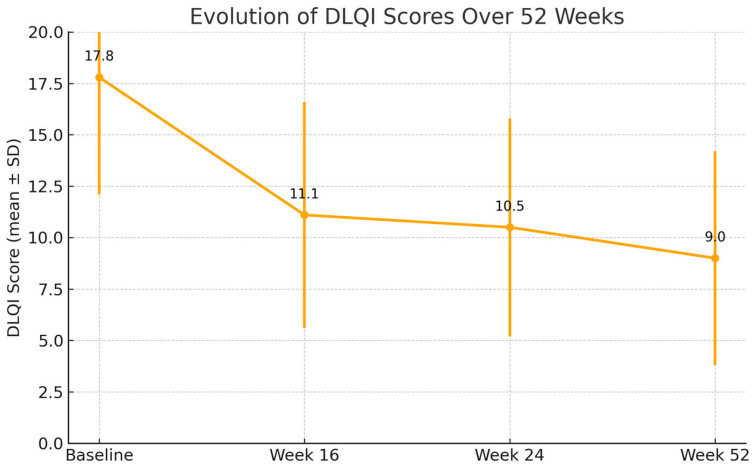
Evolution of DLQI (Dermatology Life Quality Index) over 52 weeks in patients treated with tralokinumab. Error bars represent standard deviation (*p* < 0.001).

All these results are shown in Figure 1, Figure 2, Figure 3, Figure 4 and Figure 5.

All comparisons of clinical outcomes (EASI, BSA, NRS pruritus, NRS sleep, and DLQI) between baseline, week 16, week 24, and week 52 showed statistically significant improvements (*p* < 0.001 for all parameters). Pairwise analyses confirmed significant reductions between baseline and each subsequent time point (*p* < 0.001) and further improvements between week 16 and week 52 (*p* < 0.01).

### 3.3. Clinical Response over Time Measured by EASI 75/90/100

**Figure 6:**
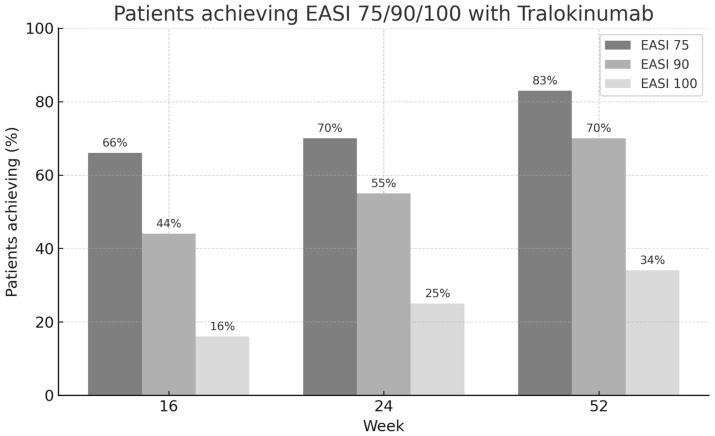
Percentage of patients achieving EASI-75, EASI-90, and EASI-100 responses at weeks 16, 24, and 52 after initiating tralokinumab treatment.

At week 16, 66% of patients achieved an EASI-75 response, while 44% reached EASI-90 and 16% achieved complete skin clearance (EASI-100). By week 24, these proportions increased to 70% for EASI-75, 55% for EASI-90, and 25% for EASI-100. At week 52, the clinical response continued to improve, with 83% of patients reaching EASI-75, 70% achieving EASI-90, and 34% attaining EASI-100 (Figure 6). These data highlight a progressive improvement in disease severity over time with tralokinumab treatment, with substantial proportions of patients achieving high levels of clinical response, including complete clearance in more than one-third of patients by one year.

When comparing treatment responses according to prior exposure to biologic therapies, outcomes were similar between biologic-naïve and biologic-experienced patients. EASI-75 responses at week 16 were 67% in biologic-naïve vs. 61% in biologic-experienced patients (*p* = 0.48), with similar differences observed at weeks 24 and 52 indicating comparable efficacy between groups.

### 3.4. Treatment Persistence and Safety

**Figure 7:**
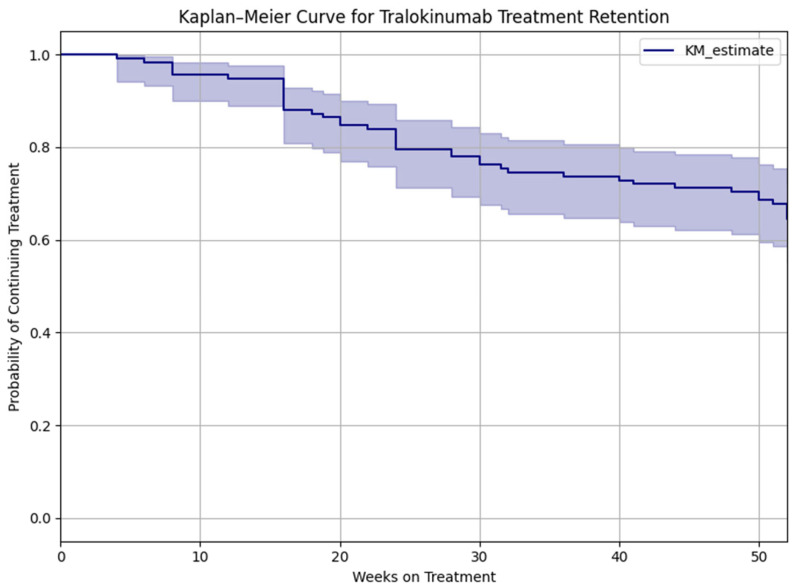
Kaplan–Meier survival curve showing the probability of continuing treatment with tralokinumab over 52 weeks. The shaded area represents the 95% confidence interval.

Regarding treatment persistence, a Kaplan–Meier analysis was performed (Figure 7) to estimate drug survival over the 52-week follow-up. The survival curve showed that approximately 70% of patients remained on tralokinumab at one year. Most discontinuations occurred within the first 16 weeks, stabilizing thereafter. The primary reasons for treatment discontinuation were lack of efficacy (23/109; 21%), followed by adverse events (2/109; 1.8%), patient preference (including pregnancy planning and complete clearance), and loss to follow-up.

Regarding safety, only three patients experienced conjunctivitis (in two cases it led to treatment discontinuation), and three patients presented with transient facial erythema. No other adverse events such as herpes infections or other types of infections were observed.

## 4. Discussion

This real-world study provides relevant data on the effectiveness and safety of tralokinumab in adults with moderate-to-severe atopic dermatitis (AD) in routine clinical practice. Our findings confirm and expand on the results of pivotal trials and contribute valuable real-world evidence (RWE) supporting tralokinumab as a therapeutic option in a heterogeneous patient population. The high proportion (78%) of biologic-naïve patients initiating tralokinumab is explained by our regional health service policy, which prioritized tralokinumab over dupilumab as first biologic therapy during the study period based solely on economic criteria without direct input from dermatologists.

One of the most remarkable findings of our study is the early and strong response to tralokinumab observed at week 16. Patients showed a mean EASI reduction of over 15 points and a rapid achievement of treatment targets: 66% reached EASI-75 and 44% achieved EASI-90. These results are notably higher than those reported in the phase 3 ECZTRA 3 trial, where 38.9% of patients receiving tralokinumab plus topical corticosteroids (TCSs) achieved EASI-75 at week 16 [6]. We think that our better results are due to the predominance of biologic-naïve patients and the optimization of treatment in our real-world setting, including the concomitant use of topical corticosteroids and close follow-up in specialized clinics. This early effectiveness is particularly relevant in clinical practice, where a rapid response is often associated with improved adherence and patient satisfaction.

Moreover, our long-term data show sustained improvement as by week 52, 83% of patients achieved EASI-75, 70% EASI-90, and 34% EASI-100. These outcomes exceed those observed in pivotal phase III trials ECZTRA 1 and 2 of EASI-75 response from 37.6% at week 16 to 61.8% at week 52, and EASI-90 from 20.4% to 37.3% [3,10]. These data confirm that tralokinumab provides not only fast but also durable disease control in patients with moderate-to-severe atopic dermatitis.

A growing bibliography of RWE [8,9,11,12,13,14,15,16,17,18,19] supports our findings. Pereyra-Rodríguez et al. found slightly lower short-term response rates, with 40% of patients reaching EASI-75 at week 16, whereas Chiricozzi et al. reported 53% EASI-75 at week 24 in a multinational cohort. Our data is similar to the recent article published by Rodriguez et al. (2025), where 93% of patients achieved IGA 0/1 after one year of tralokinumab treatment, with median BSA improving from 10.0% to 0.0% [11]. Similarly, we observed a mean reduction in BSA from 33.9% at baseline to 4.1% at week 52. Pezzolo & Naldi (2023) [18] reported rapid clinical improvement in resistant AD cases treated with tralokinumab in an open-label series. Similarly, Schlosser et al. (2023) [14] in a daily practice registry (n = 77) found EASI-75 and EASI-90 rates of 62% and 45% at week 16, consistent with our early response. De Greef et al. (2023) [15], in a multi-center prospective cohort of 21 patients, also demonstrated EASI-75 in 66.7% at week 16, with no incidents of conjunctivitis. Calabrese et al. (2024) [16] presented a large multi-center RWE study (n ≈ 150) with maintenance of response through week 52, mirroring our long-term efficacy and safety profile, including low rates of discontinuation due to adverse events. Though not directly referenced earlier, this study further validates our results from a broader European patient base.

Additional RWE publications reflect similar findings: Gargiulo et al. (2023) [17] observed rapid symptom relief and quality-of-life improvements within 16 weeks. Pezzolo et al. (2024) [18] reported high rates of treatment survival, with minimal serious adverse events after 32–52 weeks. García-Castro et al. (2024) [19] and Pereyra-Rodríguez et al. (2023) [8] each provided further support for the short-term effectiveness and favorable tolerability profile of tralokinumab.

Overall, real-world studies (Table 2) consistently show similar or even superior rates of clinical response compared to pivotal trials, with EASI-75 responses typically between 60 and 80% at week 52 and low discontinuation due to adverse events (<5%).

Drug survival is an important indicator of real-world effectiveness and tolerability of biologic therapies in chronic diseases such as atopic dermatitis. In our cohort, approximately 75% of patients continued tralokinumab treatment through week 52, reflecting a favorable long-term retention profile. This aligns with recent real-world evidence, such as the multi-center study by Pezzolo et al. which reported a similar 1-year retention rate (73%) in patients treated with tralokinumab [18]. Furthermore, Schlosser et al. also observed good persistence in a daily practice setting, with most discontinuations attributed to limited efficacy or adverse events [14]. These findings suggest that despite being a newer biologic tralokinumab demonstrates acceptable long-term adherence, comparable to or exceeding that of dupilumab in similar populations [9].

The drop-out curve in our study showed the highest rate of discontinuation within the first 4 months, consistent with other series where early dropout is often related to lack of early response or intolerance [8,9]. Nonetheless, the plateauing of the curve beyond week 24 supports that patients who respond to and tolerate the drug are likely to maintain treatment over time. These retention dynamics emphasize the importance of early response monitoring and patient education to optimize long-term outcomes.

Regarding safety, our findings reinforce the favorable profile of tralokinumab. Only two patients (1.8%) discontinued treatment due to adverse events (AEs), and only three cases of conjunctivitis were reported. Notably, the absence of conjunctivitis in the De Greef cohort [15] suggests that this side effect may be infrequent in routine care. Although our series reported a low incidence of adverse events, we acknowledge that other studies and post-marketing reports have described rare but serious complications associated with tralokinumab use, including corneal perforation, peripheral ulcerative keratitis (PUK), and severe upper respiratory tract exacerbations. Clinicians should therefore monitor patients closely, particularly regarding ophthalmologic symptoms, and consider baseline and follow-up eye assessments when appropriate [20,21].

Our discontinuation rate due to AEs was lower than that reported in clinical trials (4–6%), suggesting a favorable safety profile in real-world clinical settings. Treatment adherence and patient selection may contribute to the observed differences between studies.

This low incidence aligns with the data from ECZTRA trials and RWE studies [6,11,12]. Notably, no herpes infections or serious AEs were observed, consistent with other reports that highlight the low immunosuppressive risk associated with IL-13 inhibition [12].

An additional strength of our study is the detailed analysis of comorbidities. Asthma (53.2%), allergic rhinitis (63.3%), and food allergies (18.3%) were frequent in our cohort, reflecting the type 2 inflammatory profile common in moderate-to-severe AD. Our findings are consistent with previous data reporting high prevalence of type 2 comorbidities in patients with AD [22,23].

Importantly, 78% of our patients were naïve to advanced therapies, yet 22% had prior exposure to biologics or JAK inhibitors. This mix enhances the external validity of our results and supports the effectiveness of tralokinumab regardless of treatment history, as also suggested by Rodriguez et al. who reported similar efficacy in biologic-experienced and naïve patients [11] as in our cohort.

Our study’s strengths include a sizable multi-center cohort (N = 109), extended follow-up to 52 weeks, and a mixture of biologic-naïve and biologic-experienced patients—enhancing the generalizability of results. However, the retrospective design introduces risks of selection bias and missing data. Additionally, our safety analysis is descriptive, and the absence of a comparator restricts causal inference.

On the other hand, the retrospective design, absence of a control group, and the moderate sample size limit the generalizability of our findings.

Additionally, our center is part of the multi-center TRACE study, which is currently underway. While demographic and clinical characteristics of the TRACE cohort have been described in recent publications [24,25], real-world effectiveness outcomes are still pending and will provide valuable complementary data once available.

## 5. Conclusions

This multi-center, real-world study suggests that tralokinumab could potentially be applied in routine practice for moderate-to-severe AD. Over a 52-week period we observed substantial improvements in clinical severity scores (EASI, IGA, and BSA), patient-reported outcomes (pruritus, sleep, and DLQI), and disease control, with a favorable safety profile and high persistence rates. These results are consistent with, and in many cases exceed, those observed in pivotal trials and other real-world evidence studies. Our data support the incorporation of tralokinumab into routine clinical practice as a long-term treatment for moderate-to-severe AD, especially in patients with a high burden of disease and comorbidities. However, our results should be interpreted as preliminary given the limited sample size and lack of a control group, and further prospective studies are needed.

## Figures and Tables

**Table 1:** Baseline demographic and clinical characteristics of patients (N = 109).

Variable	Value
**Age; mean years (SD)**	39.4 (16.6)
**Sex (M:F)**	69 (63.3%); 40 (36.7%)
**Age at diagnosis; %, (n)**	Childhood: 68.8% (75)
	Adult: 31.2% (34)
**Comorbidities; %, (n)**	Obesity: 16.5% (18/109)
	Rhinitis: 63.3% (69/109)
	Conjunctivitis: 22% (24/109)
	Asthma: 53.2% (58/109)
	Nasal polyposis: 1.8% (2/109)
	Alopecia areata: 2.75% (3/109)
	Eosinophilic esophagitis: 0% (0/109)
	Food allergy: 18.3% (20/109)
	Dyslipidemia: 21.1% (23/109)
	Diabetes: 5.5% (6/109)
	Hepatitis: 2.75% (3/109)
	Cardiovascular disease: 2.75% (3/109)
	HIV: 0.9% (1/109)
	History of malignancy: 3.7% (4/109)
**Phenotype; %, (n)**	Classic: 74.3% (81/109)
	Generalized: 25.7% (28/109)
**Anatomical locations; %, (n)**	Head and neck: 67.9% (74/109)
	Hands: 31.2% (34/109)
	Prurigo: 12.8% (14/109)
**Previous treatments; %, (n)**	Cyclosporine: 94.5% (103/109)
	Methotrexate: 34% (37/109)
	Azathioprine: 11.9% (13/109)
	Phototherapy: 46.8% (51/109)
	Biologic therapy naïve: 78% (85/109)
	Dupilumab: 12.8% (14/109)
	Upadacitinib: 3.67% (4/109)
	Baricitinib: 4.6% (5/109)
	Abrocitinib: 0.9% (1/109)
**Disease severity at baseline; mean (SD)**	EASI: 24.5 (11.4)
	IGA: 3.2 (0.7)
	BSA: 33.9 (19.7)
	NRS Pruritus: 7.1 (2.2)
	NRS Sleep: 5.2 (2.2)
	DLQI: 17.8 (5.7)

Data presented as mean (standard deviation) for continuous variables and percentage (n/N) for categorical variables. Eczema Area and Severity Index (EASI), Investigator’s Global Assessment (IGA), Body Surface Area (BSA) involvement, Numeric Rating Scale (NRS) for pruritus and sleep disturbance and Dermatology Life Quality Index (DLQI).

**Table 2:** Summary of main real-world evidence (RWE) studies on tralokinumab in atopic dermatitis to compare with our series.

Study/Author	Country/Design	n	EASI-75/90/100 (%)	Discontinuation (%)	Adverse Events	Notes
Pereyra-Rodríguez et al. (2023) [8]	Spain, multi-center	45	40/NR/NR (W16)	NR	None serious	Good safety profile
Chiricozzi et al. (2023) [9]	Italy and multinational	97	NR/NR/NR 53/NR/NR (W24)	NR	NR	Multi-center European cohort
Schlosser et al. (2023) [14]	Netherlands, registry	77	62/45/NR (W16)	NR	Mild conjunctivitis	Prospective registry
De Greef et al. (2023) [15]	Belgium, prospective	21	66.7/NR/NR (W16)	0	None	No conjunctivitis reported
Calabrese et al. (2025) [16]	Multicenter Europe	136	68.5/33.3/NR	Low	Favorable safety	comparable to our cohort
Gargiulo et al. (2023) [17]	Italy	26	NR (W16)	0	None serious	Rapid subjective improvement
Pezzolo et al. (2024) [18]	Italy, multi-center	105	NR (W32–52)	Very low	None serious	High treatment persistence
García Castro et al. (2024) [19]	Spain	20	NR (W16)	NR	None serious	Short-term real-life effectiveness
Rodríguez & Smith (2025) [11]	USA, single-center retrospective	41	~90/~80/NR (W52)	Very low	NR	High IGA 0/1 (93%)
Rønnstad et al. (2025) [12]	Systematic review	811	~63/40/10 (W16, mean)	5.6	Mild conjunctivitis	12 studies included

Summary of published real-world evidence (RWE) studies evaluating the effectiveness of tralokinumab in adults with moderate-to-severe atopic dermatitis. Data include both prospective and retrospective studies with varying follow-up durations and treatment backgrounds. EASI: Eczema Area and Severity Index; NR: not reported; RWE: real-world evidence.

## Data Availability

The data supporting the findings of this study are not publicly available due to restrictions imposed by the local Ethics Committee, which prohibit data sharing outside the approved research team in order to protect patient confidentiality.

## Abbreviations

The following abbreviations are used in this manuscript:

AD	Atopic Dermatitis
IL-13	Interleukin-13
EASI	Eczema Area and Severity Index
BSA	Body Surface Area
IGA	Investigator’s Global Assessment
NRS	Numeric Rating Scale
DLQI	Dermatology Life Quality Index
RWE	Real-World Evidence
RCT	Randomized Controlled Trial
TCSs	Topical Corticosteroids
JAK	Janus Kinase
HIV	Human Immunodeficiency Virus
TRACE	Tralokinumab Real-World Clinical Experience
SD	Standard Deviation
